# Supplementary material for: Evidence-based complementary and alternative medicine conventional surgery combined with traditional Chinese medicinal retention enema for tubal obstructive infertility: A systematic review and meta-analysis
Source: PLoS One. 2023 May 18;18(5):e0285859. doi: 10.1371/journal.pone.0285859 (PMC10194984; doi:10.1371/journal.pone.0285859)
Supplement: Attachment — Submitted filename: Response to Reviewers.docx [file pone.0285859.s003.docx]

Dear Editor:

Thank you very much for allowing us to revise our manuscript. We greatly appreciate the editor and reviewers for their positive and constructive comments and suggestions on our manuscript entitled "Evidence-based Complementary and Alternative Medicine Conventional Surgery Combined with Traditional Chinese Medicinal Retention Enema for Tubal Obstructive Infertility: A Systematic Review and Meta-analysis" (PONE-D-22-35231).

In this revised version, we have addressed the concerns of the reviewers. An item-by-item response to the reviewers' comments is enclosed, and we revised the manuscript. We hope that these revisions successfully address their concerns and requirements.

Thanks again.

Point-to-point responses to the comments:

**Suggestions from editor:**

1. Please ensure that your manuscript meets PLOS ONE's style requirements, including those for file naming. The PLOS ONE style templates can be found at

https://journals.plos.org/plosone/s/file?id=wjVg/PLOSOne_formatting_sample_main_body.pdf and

https://journals.plos.org/plosone/s/file?id=ba62/PLOSOne_formatting_sample_title_authors_affiliations.pdf

2. We note that the grant information you provided in the 'Funding Information' and 'Financial Disclosure' sections do not match.

When you resubmit, please ensure that you provide the correct grant numbers for the awards you received for your study in the 'Funding Information' section.

3. Please ensure that you refer to Figure 10 in your text as, if accepted, production will need this reference to link the reader to the figure.

4. Please upload a new copy of Figure 3 and 4 as the detail is not clear. Please follow the link for more information:

**Comment:** Thank you for your suggestions. We carefully checked the format of manuscript. And we will match the information in the 'Funding Information' and 'Financial Disclosure' when we resubmit. Besides, we will upload a clearer copy of Figure 3 and 4.

**Reviewer #1:**

**Comment 1:** Concerning abstract section,

a-materials and methods, pls widen your research period till November 2022.

b- TCM stands for what? Pls define the abbreviation.

c-results, RCTs stands for, pls define

d- in all manuscript, p value should be written in italics.

**Response 1:** a: Thanks for the suggestion. We have searched other studies and checked them carefully. Unfortunately, we found that they did not meet the criteria for inclusion in the research.

b: We define TCM as a complete healing system that developed in China. We have explained it thoroughly in the manuscript. **(Line 63-69, Page5)**

c: RCTs means randomized controlled trials. **(Line 71, Page5)**

d: We have modified all p values in all manuscripts.

**Comment 2**: Introduction

a- What is tubal infertility, pls define it ?

b- The introduction is very superficial, pls add some notes about prevalence and incidence of tubal infertility in China and worldwide.

**Response 2**: a: We defined Tubal Obstructive Infertility as infertility caused by damage to the function and structure of the fallopian tubes due to pelvic infection, gynecological surgery, and other factors, causing chronic inflammatory damage and tissue fibrosis, resulting in inflammatory changes in the fallopian tubes, with pathological changes such as swelling, exudation, fluid accumulation, thickening, adhesion, stiffness, distortion, occlusion, and inaccessibility, leading to obstruction of sperm-egg union or delivery. **(Line 46-55, Page4)**

b: We revised the introduction and added some data about the prevalence and incidence of tubal infertility in China and worldwide. The global rate of infertility in women is approximately 15.5%, and the incidence is increasing. Tube obstruction can lead to reduced fertility among affected women, accounting for 25 to ~ 35% of all causes of infertility. In a study with 3018 infertility patients in China, we found that 23.46% of the patients had infertility due to tubal factors, especially in people over 35 years old, which is the leading cause of infertility. **(Line 42-46, Page6-7)**

**Comment 3:**Concerning inclusion and exclusion criteria, are all the studies have the same inclusion and exclusion criteria to be involved in this meta-analysis? And are there any similarities or differences between these studies?

**Response 3**:We have revised the inclusion and exclusion criteria**(Line 90-120, Page6-7)** and checked the studies in this research have the same inclusion and exclusion criteria. Some of the studies have differences, such as the surgical options, treatment time, follow-up time, retention enema time, and insertion depth. We analyze these differences in subgroup analysis. **(Table 2, Page 18-19)**

**Comment 4:** Concerning outcome, the incidence of ectopic pregnancy, are all other factors that affect ectopic pregnancy taken into consideration to be included or excluded, such as sexually transmitted diseases, pelvic surgery, endometriosis.

**Response 4**：Thanks for the suggestion. We added some exclusion criteria, such as sexually transmitted diseases and previous history of abdominal or pelvic surgery, combined with other gynecological diseases of the uterus and pelvis, such as endometriosis. Those with a combination of severe hemorrhoids, anal fissures, and other contraindications to enemas; patients with more serious medical complications and psychiatric disorders, combined with other infertility factors. **(Line 108-117, Page7)** Furthermore, we also refresh the studies in our research.

**Comment 5:** Does The data obtained from all clinical trials ,involved in this review, with the same treatment period? or are there any differences ? As different duration will affect cure rate.

**Response 5**：Thanks for the suggestion. Some of the studies involved in this review have different treatment periods. Thus we added treatment duration in the subgroup analysis. **(Table 2, Page 18-19).**

**Comment 6**:Concerning the outcome, I think all studies selected in this meta-analysis must have the same outcome measure to avoid bias. All the studies must affect Clinical pregnancy rate, Clinical total effective rate, incidence of ectopic pregnancy, The Improvement of TCM Symptoms, The improvement of TCM symptoms.

**Response 6：**Thanks for the suggestion. We consider the Clinical pregnancy rate and Clinical total effective rate to be significant indicators of efficacy. And the incidence of ectopic pregnancy is one of the manifestations of adverse pregnancy. It is not appropriate to directly use it to reflect the clinical effect. Besides, different studies have different definitions of TCM symptoms, which is why we finally deleted TCM symptoms in our research.

**Reviewer #2:**

**Comment 1:** This manuscript was designed to investigate Evidence-based Complementary and Alternative Medicine Conventional Surgery Combined with Traditional Chinese Medicinal Retention Enema for Tubal Obstructive Infertility: A Systematic Review and Meta-analysis.

Although, the manuscript is technically sound but it needs English editing.

The introduction provides appropriate background of the topic of this research. The results are presented in a bad format. The authors should tabulate the data in collective tables in a good format.

To make this study more substantial and applicable, the author may wish to add recent references specially in introduction.

**Response 1:** We sincerely appreciate the valuable comments. We have tried our best to polish the language in the revised manuscript. Besides, we adjusted the table format to ensure it can be fully presented **(Table 1, Page 11-15)**. We searched the recently published literature but unfortunately found that they did not meet the criteria for inclusion in the research.
